# Supplementary material for: Propranolol Ameliorates the Antifungal Activity of Azoles in Invasive Candidiasis
Source: Pharmaceutics. 2023 Mar 23;15(4):1044. doi: 10.3390/pharmaceutics15041044 (PMC10144167; doi:10.3390/pharmaceutics15041044)
Supplement: Supplementary file 1 [file pharmaceutics-15-01044-s001.zip › pharmaceutics-2251339-supplementary.pdf]

Supplementary Materials

Propranolol Ameliorates the Antifungal Activity of Azoles in Invasive Candidiasis

Supplementary Figures

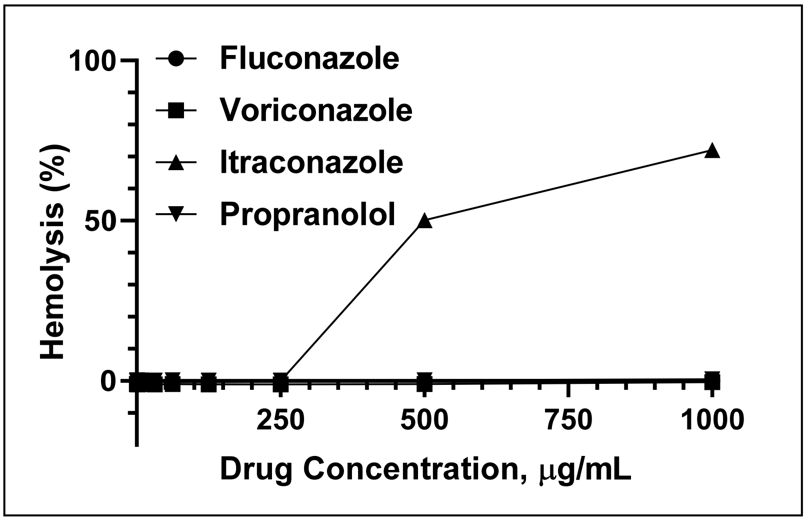

Figure S1. Haemolytic activity of propranolol and azole antifungal drugs**.**

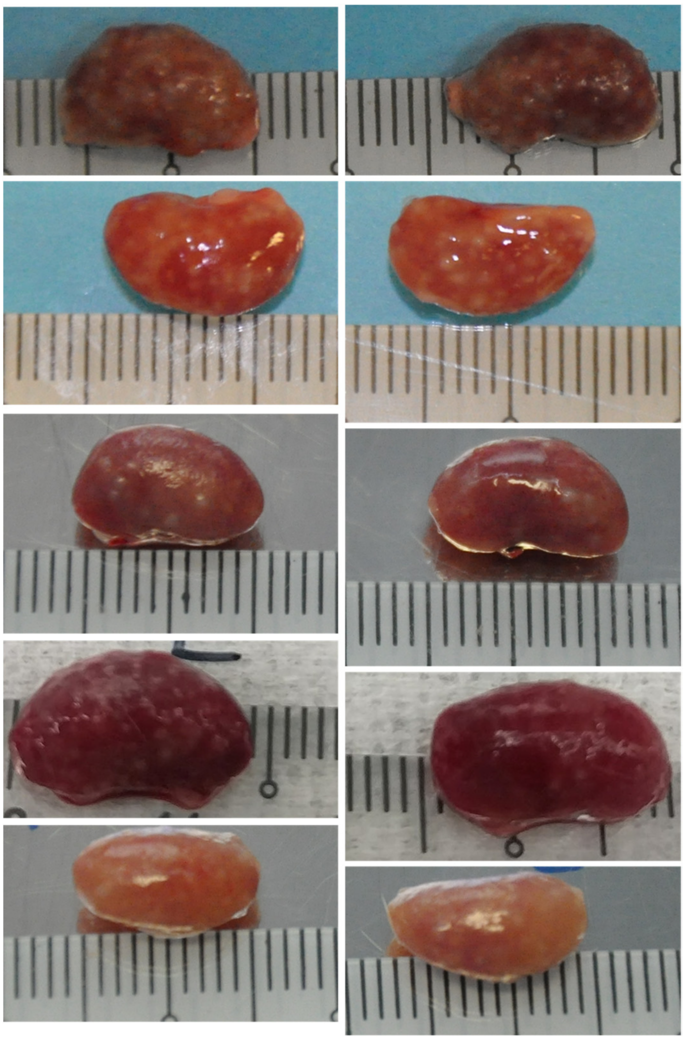

Figure S2. Representative kidney photographs of untreated control mice

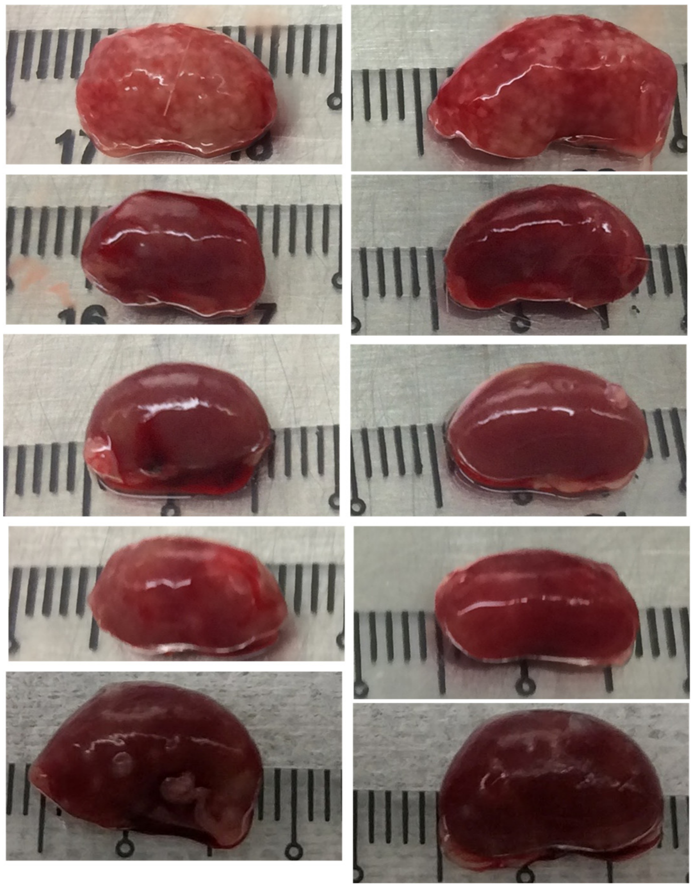

Figure S3. Representative kidney photographs of itraconazole (0.0625 mg/kg) treated mice.

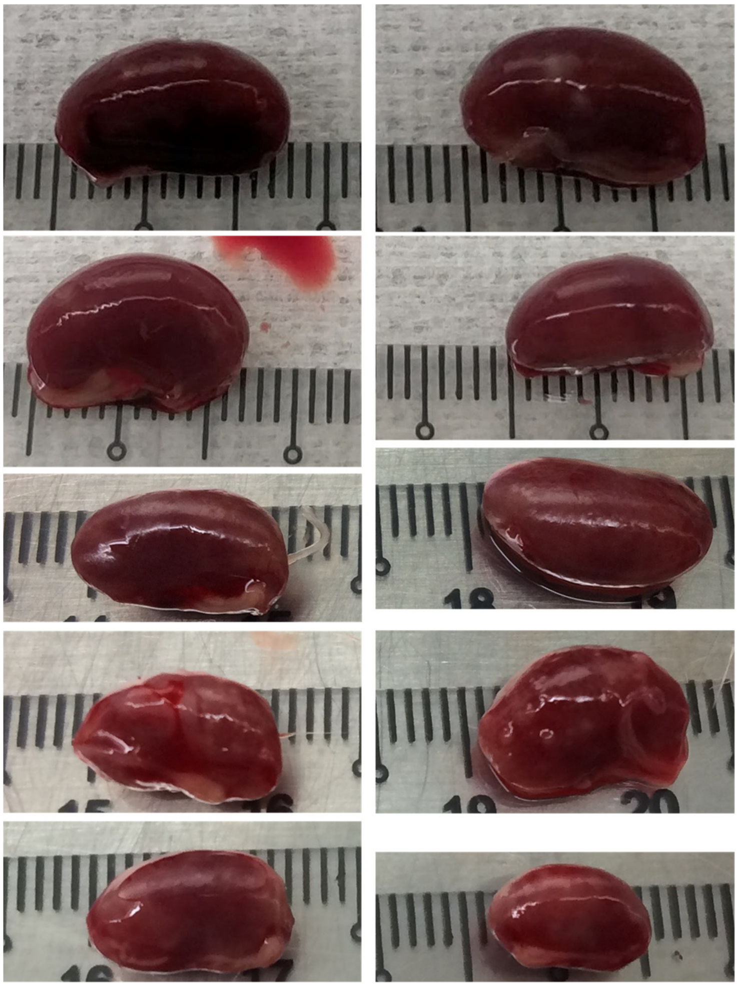

Figure S4. Representative kidney photographs of propranolol (1.25 mg/kg) treated mice.

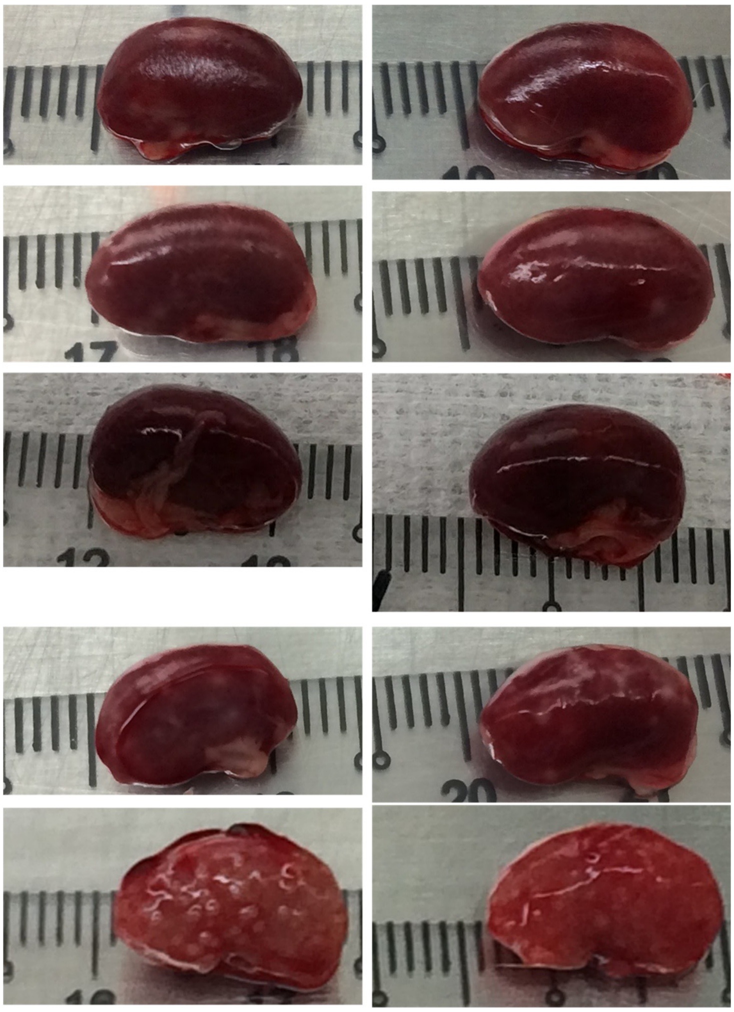

Figure S5. Representative kidney photographs of propranolol (1.25 mg/kg) and itraconazole (0.0625 mg/kg) treated mice.

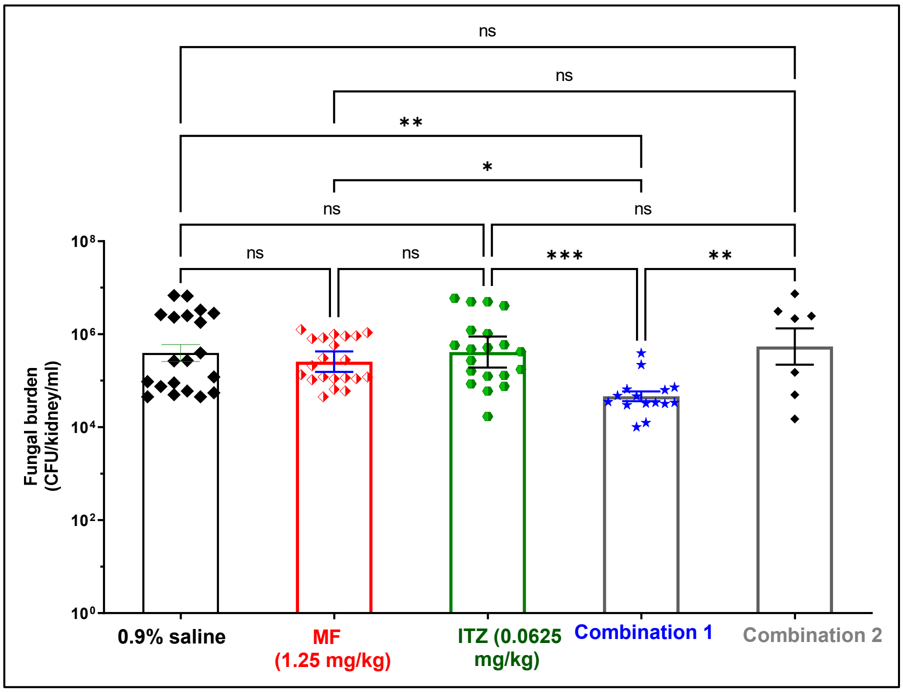

Figure S6. Fungal burden in the kidneys of mice treated with various groups (error bars indicate standard deviation). Propranolol (1.25 mg/kg) and itraconazole (0.0625 mg/kg) combination is presented as Combination 1. Propranolol (0.625 mg/kg) and itraconazole (0.0625 mg/kg) combination is presented as Combination 2.

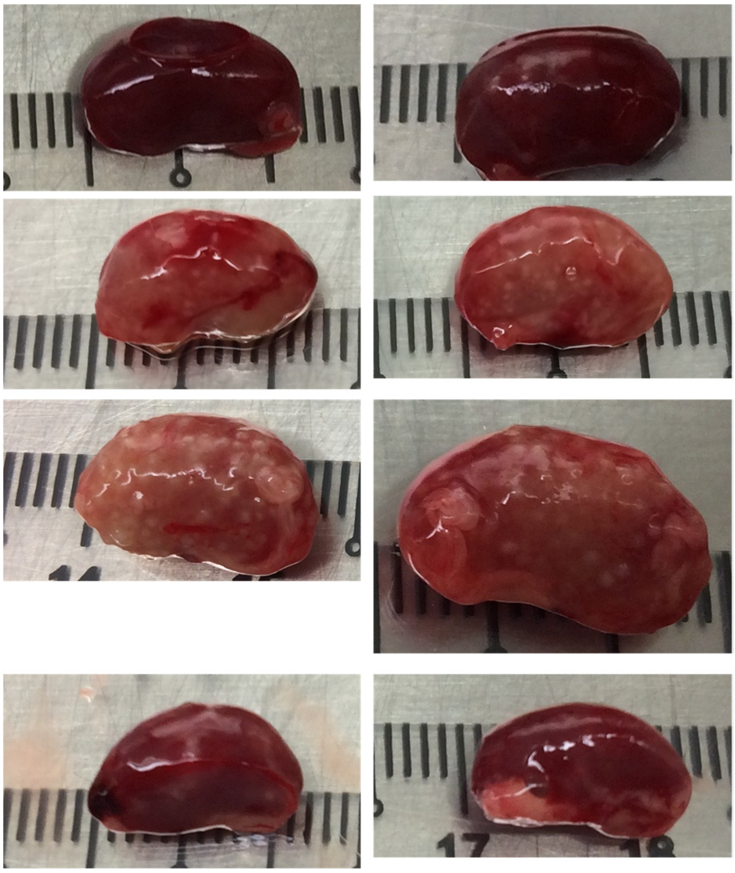

Figure S7. Representative kidney photographs of propranolol (0.625 mg/kg) and itraconazole (0.0625 mg/kg) treated mice

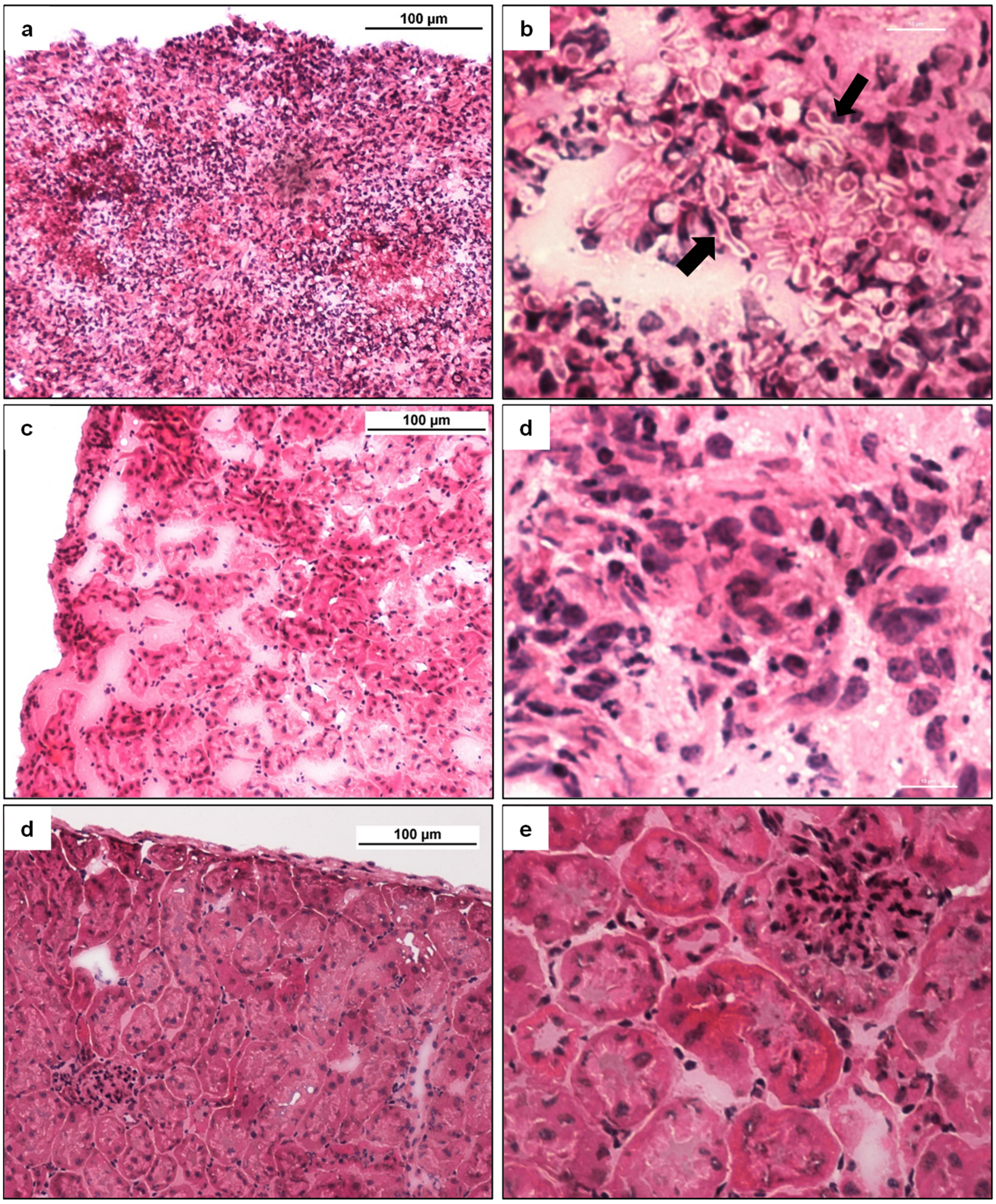

Figure S8. Histology of *C. albicans* infected mice. **(a)** H & E staining (low magnification) of kidney infected with *C. albicans* 10231 strains shows diffuse inflammation and necrosis obscuring normal renal tubule architecture. **(b)** H & E staining (higher magnification) of kidney infected with *C. albicans* 10231 strains showing *Candida* pseudohyphae (black arrow) within the inflammatory foci. **(c,d)** H & E staining of kidney infected with *C. albicans* after treatment with propranolol-itraconazole combinations showing reduced inflammation and necrosis although areas of renal tubule and glomeruli atrophy are present suggestive of a healed infection **(c)** with no obvious active fungal elements present **(d). (e,f)** H & E staining of immunosuppressed naïve mice kidney showing no active inflammation or atrophy of the kidney tubules or glomeruli. The scale bar for right panel is 10 µm.

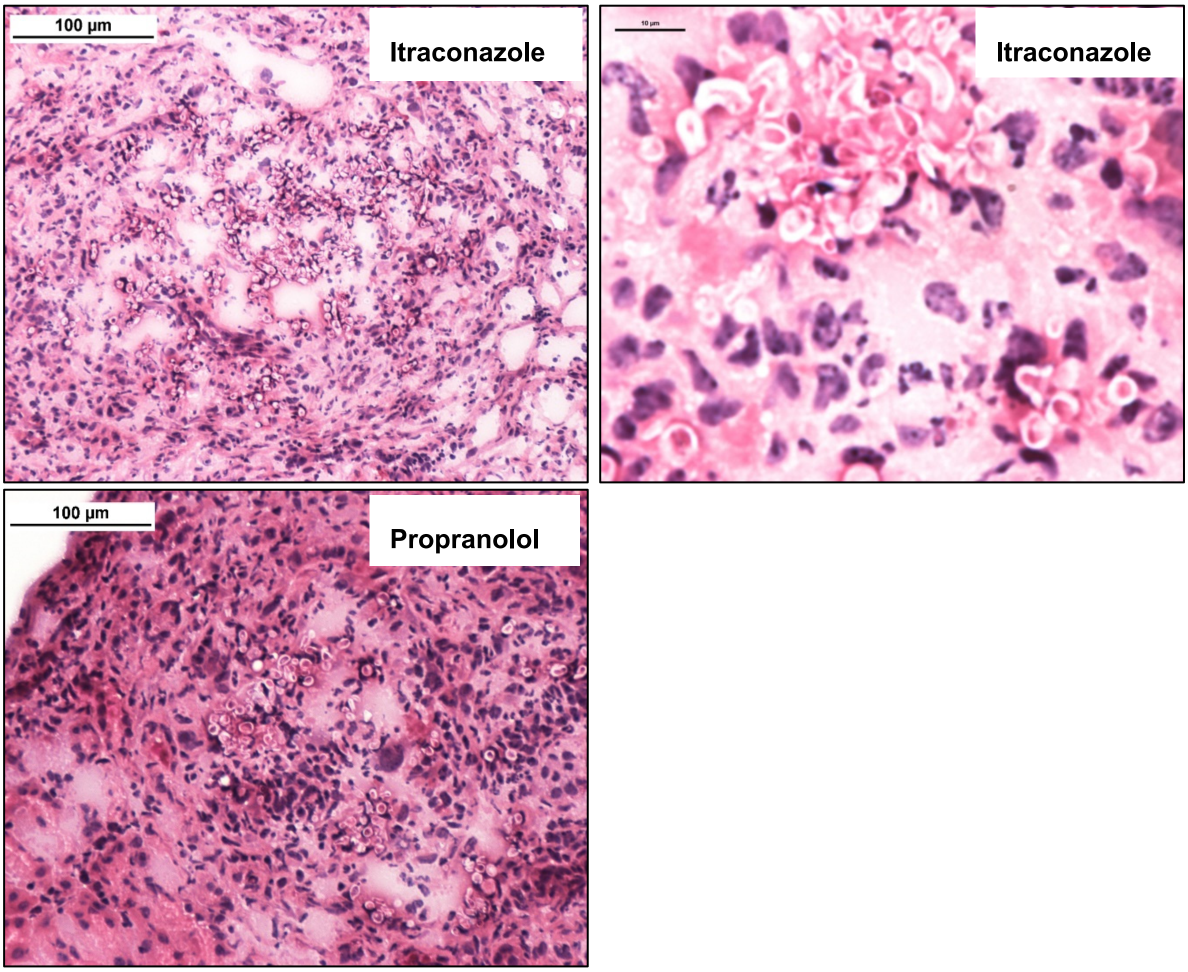

Figure S9. Histology of kidneys treated with propranolol (1.25 mg/kg) and (0.0625 mg/kg) itraconazole monotherapy.

Table S1. MIC of propranolol and other antifungal drugs against *C. albicans* (μg/mL)

Antifungal/β-blocker	*C. albicans* 10231	*C. albicans* 2672R	*C. albicans* 1976R	*C. albicans* 24433	*C. albicans* 2091
Propranolol	800	800	800	800	800
Fluconazole[a]	200/6.25	200/25	200/50	12.5/1.56	100/1.56
Voriconazole	25/<3.125	50/3.125	50/0.78	12.5/<3.125	50/0.78
Itraconazole	>25/0.78	>25/0.78	25/1.56	>25/0.78	25/1.56

[a]For azole drugs, $MIC_{80\%}/MIC_{50\%}$ values are shown which represent the minimal inhibitory concentration at 80% and 50% growth inhibition, respectively.

Table S2. Summary of the synergism between propranolol and other antifungals

Strains	$MIC_{\geq 90\%}$ (μg/mL) of azoles in the presence of 100 μg/mL Propranolol[a]		
	**Amphotericin B**	**Natamycin**	**Terbinafine**
C. albicans 1976R	NR (0.098)	NR (12.5)	6.25 (12.5)
C. albicans 2672R	0.049 (0.098)	NR (12.5)	1.56 (6.25)
C. albicans ATCC 2091	0.024 (0.049)	25 (12.5)	1.56 (3.125)
C. albicans ATCC 24433	0.049 (0.098)	NR (12.5)	1.56 (3.125)
C. albicans ATCC 10231	NR (0.098)	NR (12.5)	6.25 (12.5)

[a] Numbers in parenthesis indicate the MIC values of the antifungals.
[b] NR indicates no reduction in MIC in the presence of propranolol (μg/mL)
